# Complete sucrose hydrolysis by heat-killed recombinant *Pichia pastoris* cells entrapped in calcium alginate

**DOI:** 10.1186/1475-2859-13-87

**Published:** 2014-06-18

**Authors:** Duniesky Martínez, Carmen Menéndez, Félix M Echemendia, Enrique R Pérez, Luis E Trujillo, Alina Sobrino, Ricardo Ramírez, Yamira Quintero, Lázaro Hernández

**Affiliations:** 1 Fermentation Laboratory, Center for Genetic Engineering and Biotechnology Sancti Spíritus (CIGBSS), Circunvalante Norte S/N, Olivos 3, Apartado Postal 83, Sancti Spíritus 60200, Cuba; 2 Plant-Microbe Interactions Laboratory, Center for Genetic Engineering and Biotechnology (CIGB), Ave 31 entre 158 y 190, Apartado Postal 6162, Habana 10600, Cuba

**Keywords:** *Pichia pastoris*, *Thermotoga maritima*, Invertase, Immobilization, Alginate

## Abstract

**Background:**

An ideal immobilized biocatalyst for the industrial-scale production of invert sugar should stably operate at elevated temperatures (60-70°C) and high sucrose concentrations (above 60%, w/v). Commercial invertase from the yeast *Saccharomyces cerevisiae* is thermolabile and suffers from substrate inhibition. *Thermotoga maritima* β-fructosidase (BfrA) is the most thermoactive and thermostable sucrose-hydrolysing enzyme so far identified and allows complete inversion of the substrate in highly concentrated solutions.

**Results:**

In this study, heat-killed *Pichia pastoris* cells bearing N-glycosylated BfrA in the periplasmic space were entrapped in calcium alginate beads. The immobilized recombinant yeast showed maximal sucrose hydrolysis at pH 5–7 and 90°C. BfrA was 65% active at 60°C and had no activity loss after incubation without the substrate at this temperature for 15 h. Complete inversion of cane sugar (2.04 M) at 60°C was achieved in batchwise and continuous operation with respective productivities of 4.37 and 0.88 gram of substrate hydrolysed per gram of dry beads per hour. The half-life values of the biocatalyst were 14 and 20 days when operated at 60°C in the stirred tank and the fixed-bed column, respectively. The reaction with non-viable cells prevented the occurrence of sucrose fermentation and the formation of by-products. Six-month storage of the biocatalyst in 1.46 M sucrose (pH 5.5) at 4°C caused no reduction of the invertase activity.

**Conclusions:**

The features of the novel thermostable biocatalyst developed in this study are more attractive than those of immobilized *S. cerevisiae* cells for application in the enzymatic manufacture of inverted sugar syrup in batch and fixed-bed reactors.

## Background

The hydrolysis of sucrose generates an equimolar mixture of fructose and glucose, commercially known as invert sugar. The inverted sugar syrup is sweeter than sucrose and easier to incorporate in food and pharmaceutical preparations since it does not show the crystallization problems of its precursor in highly concentrated solutions. Sucrose inversion can be achieved by acid hydrolysis or by using invertase (EC 3.2.1.26) or exoinulinase (EC 3.2.1.80). The enzymatic process produces food-grade syrups which are devoid of the brown colour and the toxic by-product hydroxymethylfurfural (HMF) present in the acid inverted syrup. The mesophilic yeast *Saccharomyces cerevisiae* is by far the main source of enzyme for the commercial production of invert sugar. The yeast periplasmic invertase (SUC2) is a glycoprotein optimally active at pH 4.5–5.0 and 55–60°C, but its activity in immobilized biocatalysts drops drastically during repeated operation at temperature above 50°C [1-3].

Entrapment in insoluble calcium alginate gel is recognized as a simple, inexpensive, and non-toxic method for immobilization of enzymes and cells with applications in the food and pharmaceutical industries [4]. Several authors have entrapped partially purified SUC2 or *S. cerevisiae* cells in alginate gels for continuous production of invert sugar [5-9]. Immobilization of the whole yeast cells offers economic advantages over immobilization of soluble invertase. The use of immobilized cells prevents the enzyme from leaking out of the Ca-alginate beads, but may cause technical troubles during operation if the confined yeast remains alive. The sucrose fermentation products ethanol and acetate are undesired contaminants in the invert syrup while the release of CO_2_ bubbles increases the internal pressure of packed-bed columns which then tend to crack [10]. The entrapped cells can be killed with retention of the invertase activity by exposing the beads to gamma-irradiation [11], but this method is costly and not recommended for use in the food industry. Alternatively, a heat-killing process applied to *S. cerevisiae* would inactivate its thermolabile invertase.

The search for invertase or exoinulinase enzymes capable to operate at pasteurisation temperature (60-70°C) has been conducted in bacteria [12], yeast [13,14] and fungi [15-18]. *Thermotoga maritima* β-fructosidase (BfrA) is the most thermoactive and thermostable sucrose-hydrolysing enzyme so far identified. BfrA has been produced intracellularly in *Escherichia coli*[12,19] and as a secreted enzyme in *Pichia pastoris*[20]. The non-saccharolytic yeast *P. pastoris* is a Generally Recognized As Safe (GRAS) host appropriate to produce recombinant enzymes with applications in the sugar and food industries [21-23]. BfrA secretion by recombinant *P. pastoris* resulted in high invertase activity both in the periplasmic space and the growth medium offering the dual possibility of cell and enzyme immobilization [20,24].

This study is aimed to develop a thermostable biocatalyst based on the immobilization of non-viable cells for the enzymatic production of invert sugar. Heat-killed *P. pastoris* cells containing recombinant BfrA in the periplasmic space were entrapped in calcium alginate beads and compared with the free cells in terms of optimal conditions for activity, catalytic properties, and thermal stability. The immobilized biocatalyst completely hydrolysed cane sugar in highly concentrated solutions operating at high temperatures in batch and fixed-bed reactors.

## Results and discussion

### Exponential fed-batch fermentation of recombinant *Pichia pastoris* enhanced periplasmic retention of N-glycosylated BfrA

**Figure 1:**
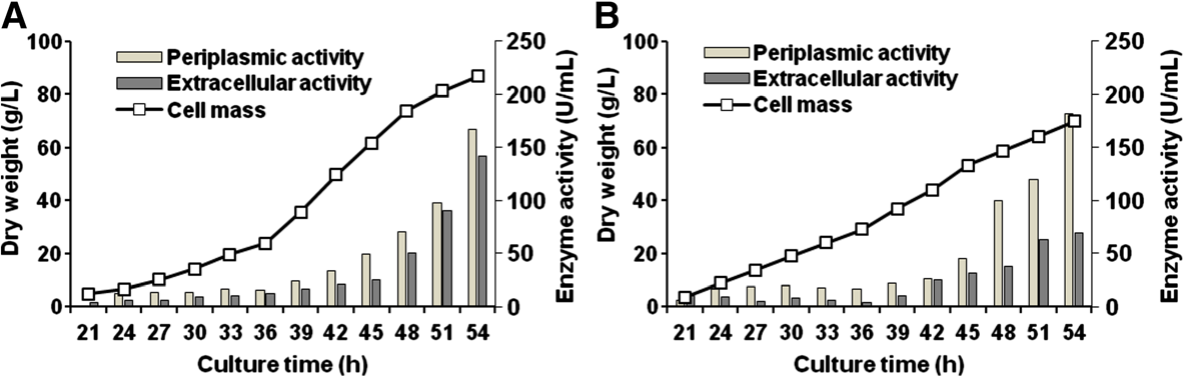
**BfrA production by recombinant *Pichia pastoris *** **strain PpBfrA (4x) during lineal (A) and exponential (B) fed-batch fermentation.**

*Pichia pastoris* strain PpBfrA (4×) constitutively expressing four copies of the *Thermotoga maritima bfrA* gene was assayed for production of the recombinant enzyme in fed-batch fermentation experiments conducted for 54 h using lineal and exponential feeding of cane sugar as the sole carbon source (Figure 1). At the end of the lineal fed-batch fermentation, the cell mass reached 84 g/L (dry biomass) and BfrA was secreted to the periplasmic space (54%) and the culture medium (46%) with a global volumetric productivity of 5735 U/L/h. The sum of the invertase activities in the undisrupted biomass (1995 U/g, dry weight) and the culture supernatant (142.1 U/mL) provides an overall BfrA yield of 309.7 U per mL of fermentation culture. The change to exponential feeding caused a reduction in the final values of cell density (70 g/L, dry biomass), extracellular invertase activity (69.6 U/mL), overall BfrA yield (251.8 U/mL) and volumetric productivity (4662 U/L/h). By contrast, the specific activity of the dry biomass (2603 U/g) increased 1.3 fold. The periplasmic/extracellular rate of invertase activity improved from 1.17 in the linear feeding to 2.61 in the exponential one. Recombinant *P. pastoris* cells grew on sucrose by a respiratory route achieving yield coefficients of 0.60 and 0.47 g dry biomass/g of sucrose for the lineal and exponential feeding strategies, respectively.

**Figure 2:**
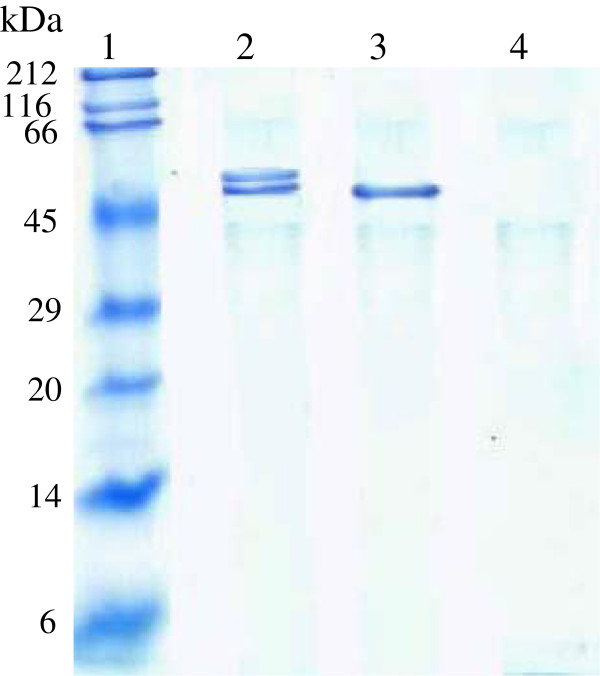
**N-glycosylation analysis of BfrA retained in the periplasmic space of recombinant *P. pastoris*.**

BfrA was extracted from the cell periplasm with purity above 90% by using a mild chemical treatment. As we have already reported for the enzyme purified from the culture supernatant [20], periplasmic BfrA migrated on SDS-PAGE as two bands of different N-glycosylation degrees with estimated molecular masses of 58 and 53 kDa (Figure 2, lane 2). The treatment with endoglycosidase H_f_ produced a deglycosylated protein with the expected size of 51 kDa (Figure 2, lane 3).

*Pichia pastoris* bearing periplasmic BfrA hydrolysed sucrose without the need of a cell permeabilization treatment, confirming that the substrate diffuses readily through the cell wall. Direct sucrose conversion in the periplasmic space has been reported for non-permeabilized cells of native *S. cerevisiae*[25] and recombinant *P. pastoris* secreting sucrose-modifying enzymes of different origins [26-28].

### Calcium alginate entrapment of heat-killed cells

Sucrose inversion by immobilized living yeast cells occurs with simultaneous substrate fermentation, which causes technical troubles as well as a decrease in the yield and quality of the inverted syrup. In this study, recombinant *P. pastoris* cells bearing the highly thermostable BfrA were submitted to a heat-killing process prior to calcium alginate entrapment. The invertase activity of the non-viable cells (3092 U/g, dry weight) increased almost 1.2-fold in comparison to the untreated cells (2603 U/g, dry weight). After biomass disruption, the soluble extracts of the viable and heat-killed cells showed no differences in their BfrA activity (3189 U/g, dry weight). Our findings suggest that heat exposition altered the cell wall structure in a way that facilitates sucrose diffusion to the periplasmic space. An alternative treatment with 70% ethanol for 15 min at 30°C succeeded to kill the *P. pastoris* cells but inactivated the periplasmic BfrA (data not shown).

**Figure 3:**
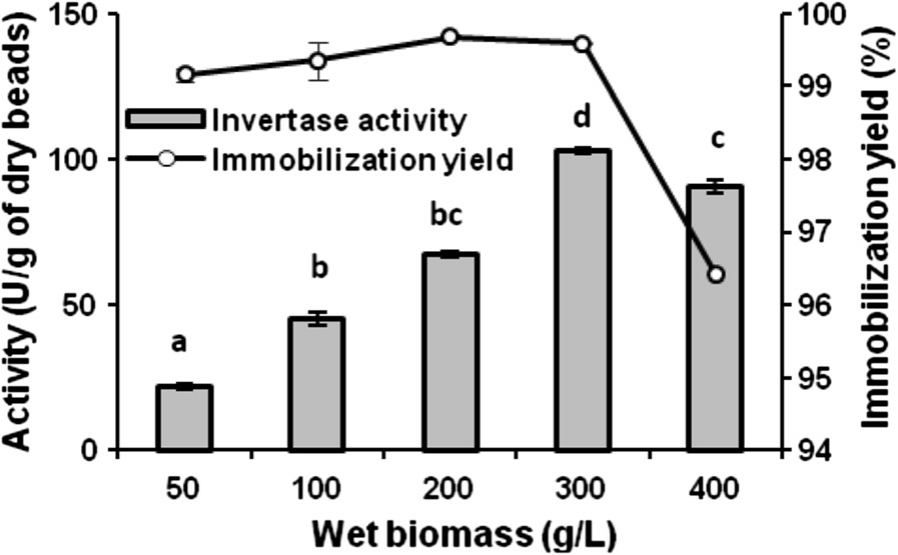
**Effect of biomass loading on the cell entrapment efficiency and the invertase activity.** Non-viable cell suspensions at final concentrations of 50, 100, 200, 300, and 400 g/L (wet biomass) were subjected to calcium alginate entrapment. The beads invertase activity was determined for sucrose (120 mM) hydrolysis at pH 5.5 and 60°C using the DNSA method. Immobilization yield expresses the percentage of initial invertase activity which was not recovered in the entrapped cells but remained in the CaCl_2_ solution. Data are the means of triplicate measurements ± standard deviation. Different letters are significantly differences between invertase activity for each quantity of biomass loading (Student-Newman-Keuls test, α = 0.05).

Figure 3 shows the effect of biomass concentration (50–400 g/L, wet weight) on calcium alginate entrapment of the heat-killed cells. Maximal values of immobilization yield (99.6%) and invertase activity (102.9 U/g of dry bead) were achieved with biomass loading of 300 g/L. The immobilization of the most concentrated biomass (400 g/L) restricted the internal diffusion of the substrate sucrose resulting in calcium-alginate beads of lower specific activity (90.7 U/g of dry bead). Cell entrapment of *S. cerevisiae* or *Bacillus macerans* at high biomass/alginate ratios did not favour the increase of intracellular invertase activity [29,30].

The entrapped cells retained 100% and 80% of its original invertase activity during storage in 1.46 M sucrose at pH 5.5 and 4°C for 6 and 12 months, respectively. The cold storage of the Ca-alginate beads in a highly concentrated sucrose solution avoided microbial contamination and allowed no important substrate dilution upon operation. Calcium alginate entrapment of free BfrA recovered from the culture supernatant resulted in a low immobilization yield and most of the entrapped enzyme leaked out of the beads during storage (data not shown).

### Effects of pH and temperature on BfrA activity in free and immobilized cells

**Figure 4:**
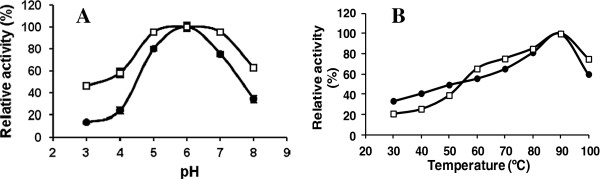
Effect of pH (A) and temperature (B) on BfrA activity in free (•) and immobilized (□) cells.

The effect of pH on sucrose hydrolysis by the non-viable *Pichia* cells in free or immobilized form was evaluated in the range 3–8 at 60°C (Figure 4A). The invertase activity of the free cells was maximum at pH 6 and decreased by at least fourfold in the reactions at the pH values 3, 4 and 8. The entrapped cells showed a less pronounced pH-activity curve with maximal hydrolysis rates in the optimum pH range 5–7 and relative activities above 40% at the extreme pH values 3 and 8. Broadening of the pH profile was also observed for *S. cerevisiae* invertase after immobilization on calcium alginate [5,31] or other supports [32-34]. Cell entrapment did not markedly influence the optimal pH conditions for BfrA activity, suggesting no changes in net charge of the biocatalyst. By contrast, immobilization of this enzyme on glyoxyl-sepharose CL-4B caused an acidic shift in the pH-activity profile, with the maximum value of sucrose hydrolysis occurring at pH 5 [24]. Similarly, the type of support and the immobilization method were found to be important factors influencing the performance of *S. cerevisiae* invertase at different reaction conditions [35,36].

The effects of pH and temperature on invertase activity of free and immobilized cells were evaluated in the ranges 3-8 and 30-100°C, respectively (Figure 4). Sucrose hydrolysis by the free heat-killed cells was maximum at pH 6 and 90°C. These values are similar to those reported for non-glycosylated BfrA produced in *Escherichia coli*[12] and N-glycosylated BfrA purified from the culture supernatant of *P. pastoris* PpBfrA(4x) [20]. The entrapped cells showed a wider optimal pH range (5-7) and higher relative activities (above 40%) at the extreme pH values 3 and 8 when comparing to the pH-activity curve of the free cells (Figure 4A). Broadening of the pH profile was also observed for *S. cerevisiae* invertase afterimmobilization on calcium alginate [5,36] or other supports [37-39]. The temperature-activity curves of the free and immobilized cells were almost identical with maximal sucrose hydrolysis at 90°C and relative activity above 60% at 60-70°C, the temperature range most recommended for industrial operation (Figure 4B).

The influence of temperature on the invertase activity of free and immobilized cells was examined in the range 30-100°C with the pH value fixed at 5.5 (Figure 4B). The temperature-activity curves of the free and entrapped cells were almost identical with maximal sucrose hydrolysis at 90°C. The immobilized biocatalyst behaved threefold less active when the reaction temperature was set at 50°C, but its relative activity remained above 65% for the reactions at 60 and 70°C, which are the most recommended temperatures for operation in the sugar industry. Optimum temperature values between 80-90°C were previously reported for the non-glycosylated BfrA produced in *Escherichia coli*[12] and the N-glycosylated enzyme in free or immobilized form [20,24].

### Effect of cell entrapment on BfrA thermal stability

**Figure 5:**
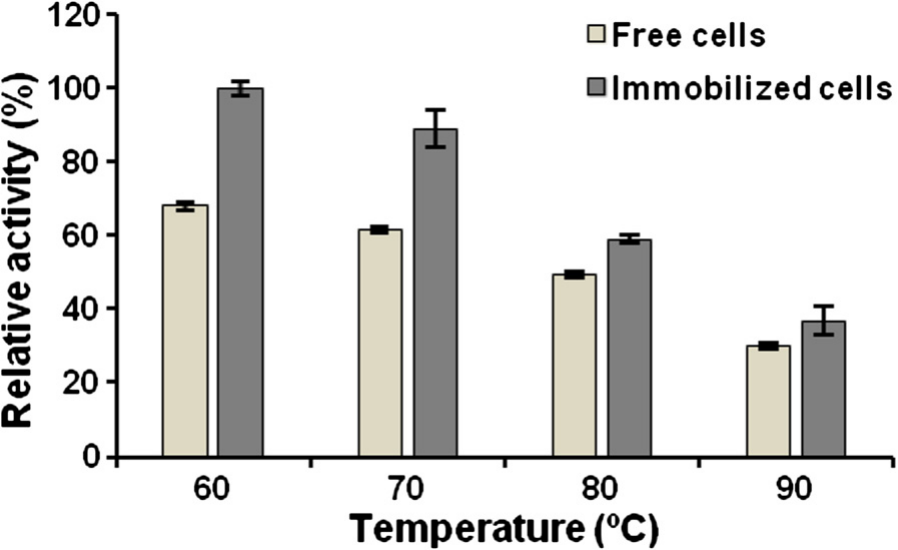
**Thermal stability of periplasmic BfrA in free and entrapped cells.** Samples were incubated in 50 mM sodium acetate buffer (pH 5.5) without substrate for 15 h at different temperatures. The remaining invertase activity was quantified by adding sucrose to 120 mM and reacting for 15 min at 60°C. Percentage values are relative to the activity of the free and immobilized cells kept at 4°C before the reaction. Data are the means of triplicate measurements ± standard deviation.

The immobilized cells retained 100, 89, 59 and 37% of the initial BfrA activity after incubation without substrate for 15 h at 60, 70, 80 and 90°C, respectively (Figure 5). The remaining invertase activity of the free cells dropped at a higher rate for all the tested temperatures. Calcium alginate encapsulation provides a physical barrier that protects periplasmic BfrA from heat inactivation and/or leakage out of the entrapped cells. Enhanced thermal stability was previously reported for *S. cerevisiae* invertase immobilized within Ca-alginate beads [7].

### Kinetic parameters and activation energy of BfrA in free and immobilized cells

N-glycosylated BfrA is known to suffer from slight inhibition at sucrose levels above 584 mM (20% w/v) [20]. The kinetic behaviour of periplasmic BfrA in free and immobilized cells was assayed using non-inhibitory substrate concentrations (10–200 mM) at pH 5.5 and 60°C. The parameter *V*_max_ was determined to be 400 μmol/min/g and 48 μmol/min/g for the free and immobilized cells, respectively. The *K*_M_ calculated for the Ca-alginate entrapped cells (136 mM) is almost 1.4-fold higher than that of the free cells (99 mM). Both *K*_M_ values exceed the one we reported previously for the free enzyme (51 mM) [20]. These findings indicate that the alginate gel and the cell wall impose a dual diffusional barrier governing the catalytic efficiency of entrapped periplasmic BfrA. Similarly, the entrapment of *S. cerevisiae* invertase in calcium alginate beads [7,31] or in polyvinyl alcohol hydrogel capsules [34] caused a reduction in the enzyme affinity for sucrose.

Activation energies for the free and entrapped cells were calculated by the Arrhenius plot according to equations *V* = 6.23 – 4441.4 (1/T) (r = 0.978) and *V* = 2.62 – 2293.9 (1/T) (r = 0.964), respectively. The activation energy of sucrose hydrolysis by the entrapped cells (19.15 kJ/mol) was lower than that displayed by the free cells (37.09 kJ/mol). This result strongly supports the assumption that BfrA reaction in the immobilized biocatalyst is limited by internal sucrose diffusion. Similar results have been reported for *S. cerevisiae* invertase immobilized on alginate or other supports [5,7,37].

### Complete sucrose hydrolysis by the immobilized biocatalyst in batch and fixed-bed bioreactors

**Figure 6:**
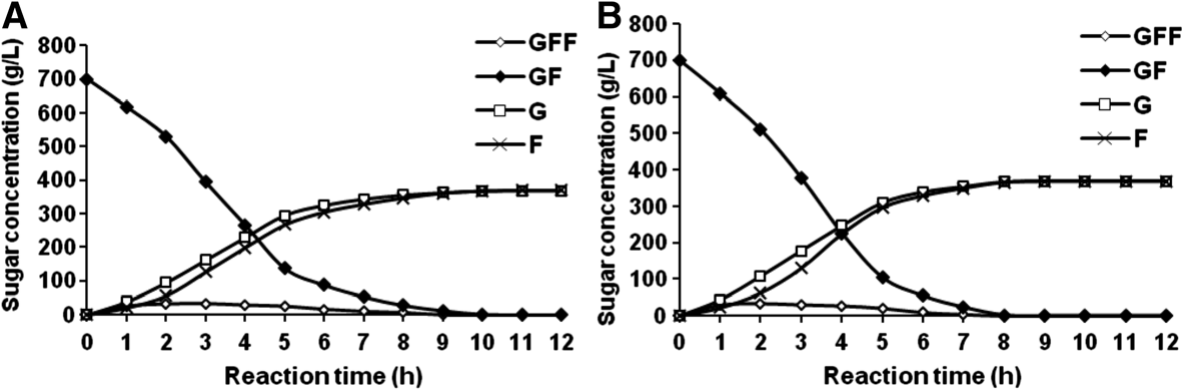
**Time course analyses of cane sugar transformation by the calcium-alginate entrapped cells.** The experiment was conducted in a laboratory-scale stirred batch reactor operating at 60 **(A)** and 70°C **(B)**. The beads (100 g, wet weight) were reacted with 2.04 M cane sugar in 50 mM sodium acetate buffer, pH 5.5 (1 L) in a stirred tank reactor. Samples (0.5 mL) were retrieved every hour and the sugar composition was determined by HPLC. The experiment was replicated three times. Standard deviation of the means was below 10%. Symbols represent F, fructose; G, glucose; GF, sucrose; GFF, 1-kestose.

Batch hydrolysis of cane sugar (2.04 M) by the entrapped cells was conducted in a laboratory-scale stirred reactor operating at 60 and 70°C. A similar product profile was observed for the time-course experiments at the two temperatures (Figure 6). The complete sucrose inversion took 8 and 10 h with average productivity of 5.43 and 4.37 gram of substrate hydrolysed per gram of dry beads per hour for the reactions at 70°C and 60°C, respectively. In both cases, the trisaccharide 1-kestose was synthesized reaching to represent above 4% (w/w) of the total sugars at time interval 1–3 h, but it was fully hydrolysed at the end of the reaction. β-fructofuranosidase enzymes commonly possess a side transfructosylating activity when sucrose concentration is above 0.3 M while at lower substrate concentrations only the hydrolytic reaction occurs [38]. The 12-h incubation at 70°C caused bead softening and promoted the occurrence of Maillard browning reactions, while the inverted sugar syrup remained colourless in the experiment at 60°C.

The continuous production of invert sugar was conducted at 60°C in a packed-bed reactor with cane sugar concentrations of 0.87, 1.46 and 2.04 M using feed flows of 30, 60 and 120 mL/h (Table 1). The highest productivity (3.5 gram of cane sugar hydrolysed per gram of dry beads per hour) was reached when the column was fed with the most concentrated solution (2.04 M) and at the fastest flow (120 mL/h). Under this operation condition, 29.7% of the initial substrate was not converted into products. The increase of the residence time by lowering the flow rate to 30 mL/h allowed the complete inversion of cane sugar at all assayed concentrations, but then the biocatalyst productivity dropped to 0.88 g/mL/h.

**Table 1:** Effects of sucrose concentration and feed flow rate on the continuous production of invert sugar

		**Hydrolysis (%)**			Productivity (g/g/h)*	
**Sucrose (M)**	**30 mL/h**	**60 mL/h**	**120 mL/h**	**30 mL/h**	**60 mL/h**	**120 mL/h**
**0.87**	100	100	95.7	0.38	0.75	1.50
**1.46**	100	99.1	76.2	0.63	1.25	2.50
**2.04**	99.8	93.6	70.3	0.88	1.75	3.50

*Productivity is expressed as gram of cane sugar hydrolysed per gram of dry beads per hour.

### Long-term stability of the immobilised biocatalyst during batch and continuous production of invert sugar

**Figure 7:**
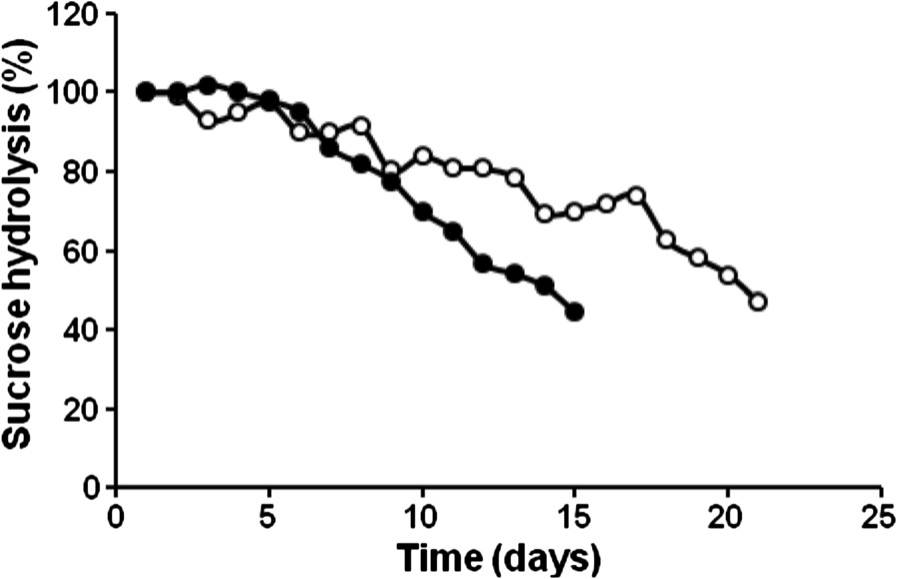
**Operational stability of the immobilized cells.** Batch and continuous reactions were conducted at laboratory scale in a stirred tank reactor (●) and a packed-bed column (○) using 2.04 M (70% w/v) cane sugar at 60°C and pH 5.5. The stirred tank was operated with 50 g of wet beads (8 g dry weight) in 1 L of substrate solution in successive batches repeated every 24 h. The packed-bed column was loaded with 150 g of wet beads (24 g dry weight) and fed at flow rate of 30 mL/h.

The operational stability of the entrapped cells was determined during batch and continuous hydrolysis of cane sugar (2.04 M) at 60°C (Figure 7). The beads retained 98%, 70%, and 47% of its original invertase activity after recycling in a stirred tank reactor for 5, 10, and 15 days, respectively. The biocatalyst behaved even more stable when operated in a packed-bed column. In this case, the beads were still 48% active after reuse for 21 days with feed flow of 30 mL/h. The half-life values of BfrA in the entrapped cells during batchwise and continuous operation at 60°C were 14 and 20 days, respectively. The lower stability of the beads in the stirred tank reactor may be attributed to the shear stress caused by agitation. The half-life of 20 days is four-fold higher than the value reported for continuous operation at 60°C of BfrA covalently immobilized on Glyoxyl-Sepharose CL 4B [24].

Immobilized biocatalysts for the enzymatic production of invert sugar are preferred to stably operate at high sucrose concentrations and pasteurization temperatures. Current biocatalysts comprising entrapped *S. cerevisiae* cells or the immobilized invertase exhibit substrate inhibition kinetics and behave rather no stable at temperature exceeding 50°C [2]. Immobilized biocatalysts using other microorganisms or enzymes have shown technical limitations such as low thermal stability, decreased catalytic efficiency, substrate inhibition, incomplete sucrose inversion, or by-products formation [2,13,14,16,30,39-41]. The immobilized whole-cell biocatalyst developed in this work fully hydrolyzed cane sugar in a highly concentrated solution (2.04 M; 70%, w/v) during repeated batches or continuous operation at 60°C without a remarkable loss of the invertase activity. The resulted colorless syrup is devoid of by-products and does not require subsequent purification or extensive concentration steps prior to commercialization. The fact of using heat-killed cells for immobilization not only avoided the occurrence of sucrose fermentation during the storage and operation periods but also prevented the enzyme from leaking out of the beads, as it did occur when free BfrA was submitted to calcium alginate entrapment. Our current efforts are directed to employ the non-viable entrapped *P. pastoris* cells bearing periplasmic BfrA as a thermostable biocatalyst for complete sucrose inversion in a packed-bed reactor at industrial scale.

## Conclusions

In this study we have developed a highly thermostable biocatalyst useful for the manufacture of invert syrup. The enzyme of choice was the exo-β-fructosidase (BfrA) from *Thermotoga maritima* secreted to the periplasmic space of *Pichia pastoris*. Heat-killed yeast cells with total retention of the invertase activity were entrapped in calcium alginate beads. The immobilized biocatalyst was successfully used at a laboratory scale for the complete hydrolysis of highly concentrated cane sugar syrup in repeated batches and continuous operation at 60°C.

## Methods

### Microorganism and fed-batch fermentation

Recombinant *Pichia pastoris* strain PpBfrA(4x) constitutively expressing four codon-optimized copies of the *Thermotoga maritima* β-fructosidase gene (*bfrA*) fused to the *Saccharomyces cerevisiae* α-factor signal sequence was used throughout this study [20]. Fed-batch fermentation was performed in a 7.5-L fermenter (INFORS) containing 3 L of growth medium [1% (w/v) cane sugar, 0.5% (w/v) yeast extract, 2.2% (w/v) (NH_4_)_2_SO_4_, 1.82% (w/v) K_2_HPO_4_, 0.75% (w/v) MgSO_4_ 7H_2_O, and 0.05% (w/v) CaCl_2_ 2H_2_O, with vitamins and traces prepared as recommended by Cregg *et al.*[42] and inoculated with 0.2 L of a shaking batch culture to an initial cell concentration of 3 g/L (wet biomass). The operation conditions during the batch phase were 30°C, pH 5.5, agitation at 500 rpm, and aeration 1 vvm. The fed batch phase started after carbon source depletion, judged by a sharp dissolved oxygen increase. The feeding medium [50% (w/v) cane sugar and 0.5% (w/v) yeast extract] was added either lineally at a constant flow of 8 mL/h/L of initial volume or exponentially according to the equation $F=\frac{\left(\mathrm{\mu XoVo}\right)}{\mathrm{Yx}/s\left(\mathrm{Sf}-\mathrm{So}\right)}\times e^{\mathrm{\mu t}}$ where F is flow rate, μ is specific growth rate (0.1 h^−1^), *Xo* is total amount of cells in the bioreactor (2.4 g dry biomass), *Yx/s* is yield coefficient (0.75 g dry biomass per gram of sucrose), *Vo* is culture volume (3 L), *Sf* is sucrose concentration of the feeding solution (500 g/L) and *So* is initial sucrose concentration in the bioreactor (10 g/L) and t is feeding time (variable parameter). During the feeding phase agitation and aeration were increased to 900 rpm and 2 vvm, respectively. Lineal and exponential fed-batch fermentations were conducted for 54 h. The biomass from the exponential fed-batch fermentation was harvested by centrifugation and used for extraction of periplasmic BfrA and cell immobilization experiments.

### Extraction of periplasmic BfrA and endoglycosidase H_f_ treatment

Yeast biomass (10 g, wet weight) was washed twice with distilled water, resuspended in 40 mL of 0.1 M NaHCO_3_ and incubated with shaking (120 rpm) for 1 h at 37°C. After centrifugation at 10,000 × g for 10 min, invertase activity was measured in the cell debris and the soluble extract. BfrA activity was mostly recovered (90%) in the soluble fraction and the protein purity was determined on SDS-PAGE gels by densitometric analysis. For N-glycosylation analysis, periplasmic BfrA (10 μg) was denatured in 100 μl of 0.5% (w/v) SDS, 1% (v/v) β–mercaptoethanol at 100°C for 10 min. After addition of 1/10 volume 1 M sodium citrate buffer (pH 5.5), the sample was reacted with endoglycosidase H_f_ (New England Biolabs) at 0.25 U/μg of total protein at 37°C for 5 h.

### Cell disruption

Wet biomass (1 g) was washed in distilled water and resuspended in 0.4 mL of breaking buffer [5% (v/v) glycerol, 1 mM PMSF, 1 mM EDTA, 50 mM sodium phosphate pH 6.0]. After addition of equal volume of acid–washed 500–μm glass beads (Sigma), the cells were mechanically lysed by ten cycles of vortex for 30 seconds and ice incubation for 30 seconds. The cell debris and the soluble extract fraction were separated by centrifugation and assayed for invertase activity.

### Preparation and storage of calcium alginate beads

The biomass pellet (150 g, wet weight) was washed, resuspended in deionized water (300 mL) and incubated at 70°C for 30 min to kill the cells prior to calcium alginate entrapment. The heat-killed cells in amounts of 2.5, 5, 10, 15, and 20 g (wet weight) were resuspended in distilled water (50 mL) to achieve final biomass concentrations of 50, 100, 200, 300, and 400 g/L, respectively. Sodium alginate (1 g) was added to the cell suspensions at room temperature and mixed thoroughly using a homogenizer. The alginate/cell mixtures were dropped through a fine needle into 37 mM CaCl_2_ solution (500 mL) with constant stirring (100 rpm) using an impeller type marine propeller to avoid droplet aggregation. Gelation time was restricted to one hour after which the CaCl_2_ solution was discarded. The spherical alginate beads (diameter 2–3 mm) were hardened overnight in 67 mM CaCl_2_ at 4°C and stored in 1.46 M sucrose in 50 mM sodium acetate buffer (pH 5.5) at 4°C before use. Ca-alginate entrapped cells used for biochemical characterization and operation experiments were prepared using wet biomass concentration of 300 g/L as described above.

### Enzyme assays

Ca-alginate entrapped cells (25 wet beads) or free cells (25 mg, wet weight) were reacted for 15 min at 60°C in 10 mL of 120 mM sucrose solution in 50 mM sodium acetate buffer (pH 5.5), unless stated otherwise. The reducing sugars released from sucrose hydrolysis were quantified using the dinitrosalicylic acid (DNSA) colorimetric method [43]. An equimolar mixture of glucose and fructose was used for the calibration curve. One unit of invertase activity was defined as the amount of enzyme required for hydrolysis of one μmol of sucrose per minute, under the above-mentioned reaction conditions. The effect of pH and temperature on the invertase activity of free and immobilized cells was assayed in 15-min reactions with 120 mM sucrose. The kinetic parameters *K*_M_ (apparent Michaelis constant) and *V*_max_ (apparent maximum rate) of free and immobilized cells were calculated from Lineweaver-Burk plots by measuring in triplicate the initial reaction rates (Vo) with sucrose ranging 10–200 mM in 50 mM sodium acetate buffer (pH 5.5) at 60°C. The activation energy of free and immobilized cells was calculated using the Arrhenius equation after measuring enzyme activities at 60, 70, 80 and 90°C [44]. The storage stability of the biocatalyst at 4°C was evaluated every month during one year. Samples of the stored beads were washed tree times in 1.46 M sucrose in 50 mM sodium acetate buffer (pH 5.5) and assayed for invertase activity in a batch operation mode as described below.

### Batch and continuous sucrose hydrolysis

Batch reactions in time-course experiments were conducted for 12 h at 60°C and 70°C using 100 g of calcium alginate beads (wet weight) incubated with 1 L of 1.75 M cane sugar solution in 50 mM sodium acetate buffer (pH 5.5) in a tank reactor with constant stirring (100 rpm). Samples (0.5 mL) of the reaction mixture were retrieved every 1 h and the sugar composition was determined by HPLC. The stability of the immobilized biocatalyst operating batchwise was evaluated at 60°C for 15 consecutive days. In each cycle, wet beads (50 g) were reacted with 1 L of the above-mentioned substrate solution for 24 h, recovered by filtration and assayed for residual invertase activity using the DNSA method.

Continuous reactions were performed at 60°C in a 250-mL column packed with 150 g of wet beads using various cane sugar concentrations (0.87, 1.46 and 2.04 M) in 50 mM sodium acetate buffer (pH 5.5) at constant flow rates of 30, 60 and 120 mL/h. The stability of the immobilized biocatalyst in continuous operation was evaluated during 21 days of reuse at 60°C.

### Protein and carbohydrate analysis

Protein concentration was estimated by the Bradford method using bovine serum albumin (BSA) as standard [45]. SDS-PAGE was performed according to [46]. The carbohydrates resulted from the BfrA biocatalyst hydrolase and transferase reactions in a highly concentrated sucrose solution (2.04 M) were quantified by high performance liquid chromatography (HPLC) using an Aminex HPX-42C column (0.78 × 30 cm, BIORAD) equipped with a refractive index detector. The column temperature was kept at 85°C. Water was used as a mobile phase at a flow rate of 0.6 ml/min. Samples were appropriately diluted before injection. Fructose, glucose, sucrose, and 1-kestose (20 mg/ml) were used as standards.

## Competing interest

The authors declare that they have no competing interest.

## Authors’ contributions

DM carried out the Constitutive expression of the synthetic *bfr*A gene in *Pichia pastoris* using fermenters. Determined the kinetic parameters of free and immobilized recombinant invertase. Design of biocatalysts of free and immobilized cells. Manuscript draft writing and editorial handling. CM Worked in the cloning and constitutive expression of the synthetic *bfr*A gene in *Pichia pastoris.* Determination of copy number integrated into the yeast genome and periplasmic extraction of the recombinant enzyme. FME worked in the establishment of the inactivation and immobilization conditions of the cells. ERP carried out the experiments of the immobilized biocatalyst operation in batch and fixed-bed bioreactors. AS evaluated the Long-term stability of the immobilised biocatalyst during batch and continuous production of invert sugar. LET carried out the screening to get the best invertase producers *Pichia pastoris* recombinant clones for further fermentation experiments. Carbohydrate analyses by HPLC and sugars quantification. Manuscript writing and editorial handling. RR worked in the analytical support in the determination of kinetic parameters and influence of different parameters in the invertase activity of free and immobilized cells. YQ determined the cell entrapment effect on *Bfr*A thermal stability. Determination of substrate concentration effects on free and immobilized enzyme productivity of intact dead cells. LH was uencharged of the overall scientific management strategies of *bfr*A cloning and gene expression in *P. pastoris*. Biochemical characterization of the recombinant enzyme. Manuscript draft writing. All authors read and approved the final version of the manuscript.
